# A Case of Laryngeal Fracture Precipitated by Swallowing

**DOI:** 10.7759/cureus.10303

**Published:** 2020-09-07

**Authors:** Edward Balai, Navdeep Bhamra, Keshav K Gupta, Karan Jolly, James Barraclough

**Affiliations:** 1 Department of Otolaryngology, The Royal Wolverhampton NHS Trust, Birmingham, GBR

**Keywords:** non-traumatic laryngeal fracture, otolaryngology, cartilage fracture

## Abstract

We report the case of a non-traumatic laryngeal fracture precipitated by swallowing where the symptoms were initially misinterpreted as representing a possible laryngeal malignancy. By the time of diagnosis, the injury was associated with an anterior neck abscess that required urgent surgical intervention. A 61-year-old male presented with dysphonia, odynophagia and neck swelling that had begun shortly after feeling a sudden crack in his neck upon swallowing. This was initially suspected to represent a laryngeal malignancy until, while awaiting outpatient investigation, the patient re-presented with rapid progression of his symptoms. Urgent CT scan revealed a vertical fracture of the thyroid cartilage, and a large anterior neck abscess causing posterior displacement. This required urgent surgical drainage. No underlying neoplasm was found, and the patient made a full recovery with complete resolution of symptoms. Non-traumatic laryngeal fractures are extremely rare. This case demonstrates the diagnostic challenge they can pose and is the first to describe the presentation and surgical management of a case with fracture displacement due to localised infection.

## Introduction

Laryngeal fractures are uncommon, with an estimated incidence of 1 in 30,000 patients presenting to hospital [1]. However, it can be a potentially life-threatening injury and therefore requires early diagnosis and management. The majority of cases occur as a consequence of high-energy direct trauma to the neck. Non-traumatic laryngeal fractures are very rare. A total of only seven cases have been reported in the literature, with sneezing, coughing and swallowing described as the precipitating events [2-7]. While blunt trauma to the neck would prompt active and thorough assessment for a laryngeal fracture, clinicians may not be aware that this type of injury can also occur after a seemingly innocuous event. We report the case of a non-traumatic laryngeal fracture precipitated by swallowing, where the symptoms were initially mistaken to represent a possible laryngeal malignancy. By the time of diagnosis, the injury had been complicated by the development of an associated anterior neck abscess.

## Case presentation

**Figure 1:**
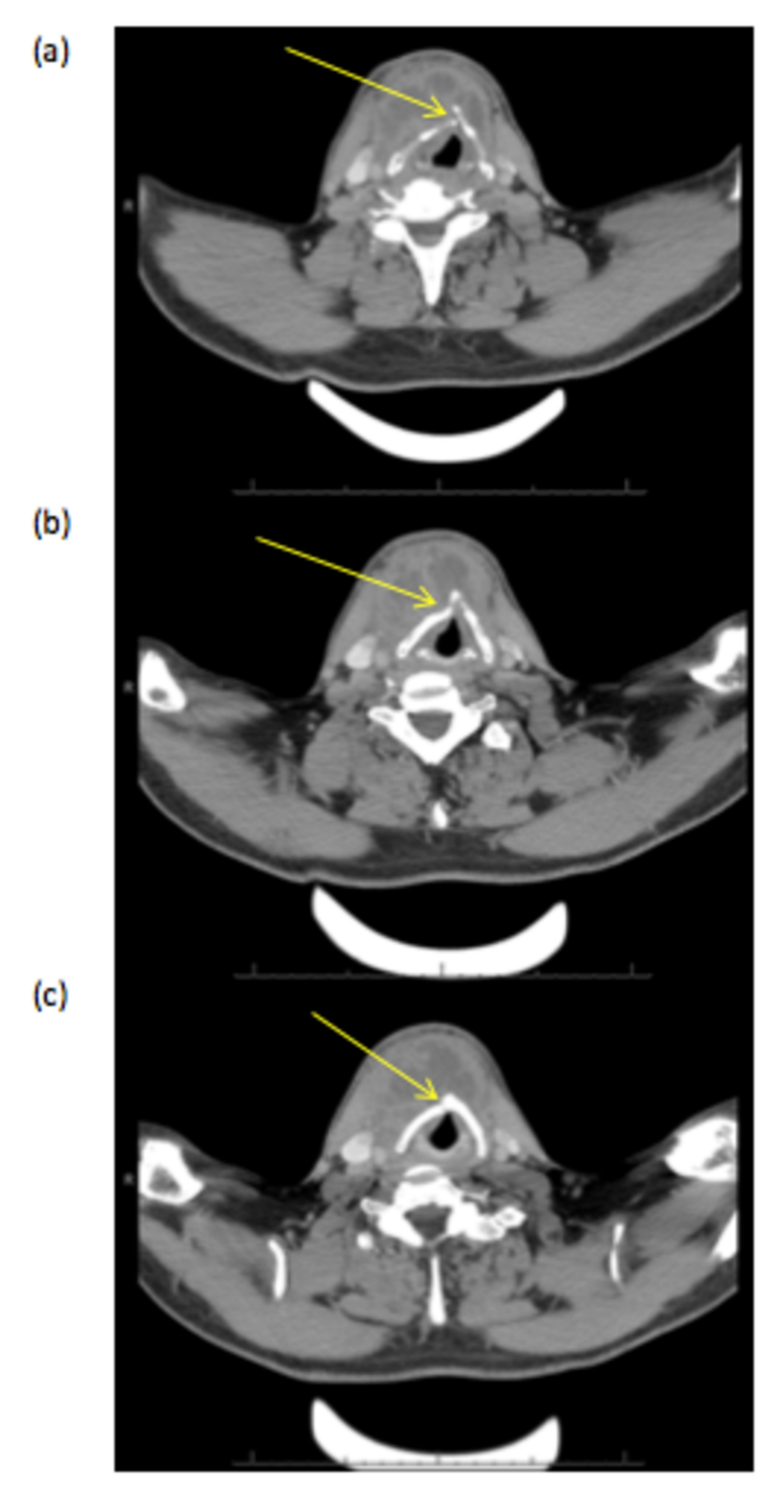
Axial sections (a-c) of contrast-enhanced CT scan demonstrating a vertical fracture of the thyroid cartilage (red arrows), with depression of the right thyroid lamina posteriorly due to anterior neck collection.

**Figure 2:**
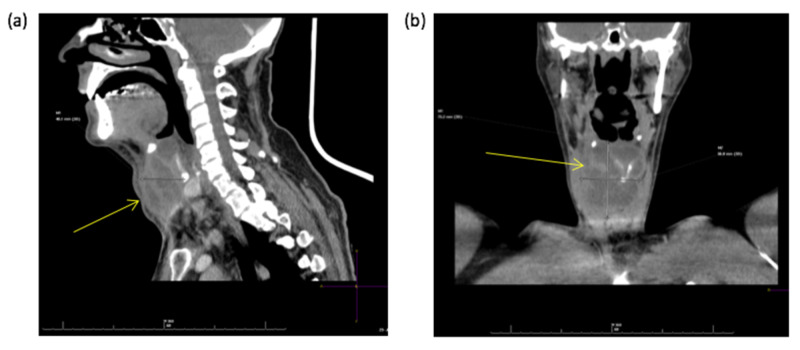
Sagittal (a) and coronal (b) sections of contrast-enhanced CT scan demonstrating a multiloculated collection (red arrows) in the midline of the anterior neck, measuring 7.5 cm × 5.6 cm × 4 cm.

A 61-year-old male, ex-smoker, with a history of type 1 diabetes, was referred by his general practitioner (GP) to the ear, nose and throat (ENT) department with odynophagia, dysphonia, dysphagia and anterior neck swelling. The symptoms began while he was eating a cracker and, upon swallowing, he felt a painful crack in the right side of his neck. By the following day, the patient had developed hoarseness and pain on swallowing. He attended his GP five days later after developing neck swelling. At this point, it was thought the symptoms may represent an infection or a possible underlying malignancy; the patient was prescribed a course of oral antibiotics and referred urgently to the head & neck clinic. At subsequent review in clinic, the patient reported ongoing odynophagia, dysphonia and dysphagia. Examination noted some fullness of the anterior neck. Flexible nasendoscopy (FNE) showed pooling of saliva and swelling of the right supraglottis. The patient was otherwise systemically well and managing to maintain adequate oral intake despite his symptoms. An expedited outpatient CT scan of the neck was requested to investigate for possible laryngeal malignancy. While awaiting this investigation, the patient re-presented to his GP with rapidly progressing symptoms and was referred to the ENT on-call team for same-day review. The patient had worsening dysphagia, now only managing sips of water. The anterior neck swelling had increased significantly in size and was now indurated with a central area of fluctuance. He underwent urgent inpatient contrast-enhanced CT scan of the neck (Figures 1, 2).

This demonstrated a vertical fracture of the thyroid cartilage to the right of the midline, with depression of the right thyroid lamina posteriorly causing mass effect on the trachea. There was also a large multiloculated collection in the midline just anterior to the thyroid cartilage. This extended superiorly up to the hyoid bone and inferiorly over the thyroid gland. The patient was commenced on intravenous antibiotics and underwent incision and drainage of the anterior neck collection. Through a lower transverse crease incision, 50 ml of frank pus was drained from the cavity and a corrugated drain left in situ. At intubation, the anaesthetist noted the patient had started to develop supraglottic oedema, and he was therefore kept intubated and ventilated overnight on the intensive care unit. Regular intravenous dexamethasone (AAH Pharmaceuticals, London, United Kingdom) was initiated postoperatively. The patient was extubated on postoperative day 1 and started on a soft diet. However, on postoperative day 2 he reported some problems with swallowing. He was kept nil by mouth for a water-soluble contrast swallow, which showed no evidence of pharyngeal or oesophageal leak. Swallowing function subsequently improved with time. The drain was removed on day 3, and the patient was discharged on postoperative day 7 to complete a short course of oral antibiotics. At one-month follow-up, there was no recurrence of symptoms. FNE examination was normal. Ultrasound of the neck confirmed complete resolution of the collection.

## Discussion

Non-traumatic laryngeal fractures are exceedingly rare. Since the first reported case in 1950 [2] there have only been six other cases described (Table 1).

**Table 1:** A summary of all previously reported cases of non-traumatic laryngeal fracture.

Case	Demographic	Precipitating event	Flexible nasendoscopy findings	CT findings	Management
Quinlan (1950) [2]	44-year-old, male	Sneezing	Supraglottic oedema. Normal vocal cord movements	N/A	Observation: anterior neck strapping, liquid diet for three days, voice rest
Martinez et al. (2007) [7]	29-year-old, male	Sneezing	Right vocal cord oedema. Right false vocal cord haematoma. Normal vocal cord movements	Anterior thyroid cartilage fracture. Non-displaced	Observation: voice rest
Alexander and Toynton (2012) [6]	41-year-old, male	Coughing	Left vocal cord haematoma. Normal vocal cord movements	Midline thyroid cartilage fracture. Slight displacement	Observation: nil by mouth for three days, voice rest
Fenig et al. (2013) [5]	47-year-old, male	Coughing	Right aryepiglottic fold and arytenoid cartilage oedema. Normal vocal cord movements	Anterior thyroid cartilage fracture. Mildly displaced. Adjacent phlegmon	Observation: voice rest, oral antibiotics
Santamaria et al. (2017) [3]	36-year-old, male	Sneezing	Left vocal cord haematoma. Normal vocal cord movements	Left parasagittal thyroid cartilage fracture. Non-displaced	Observation: voice rest, oral corticosteroids
Santamaria et al. (2017) [3]	32-year-old, male	Swallowing while bending over	Supraglottic oedema. Normal vocal cord movements	Left parasagittal thyroid cartilage fracture. Non-displaced	Observation: voice rest, non-steroidal anti-inflammatory drugs
Matrka and Li (2018) [4]	35-year-old, male	Sneezing	Right vocal cord oedema. Normal vocal cord movements	Right parasagittal thyroid cartilage fracture. Non-displaced	Observation: voice rest

While four cases described a fracture occurring after sneezing [2-4,7], and two reported it after coughing [5,6], there has only been one that mentioned a fracture occurring after swallowing [3]. In that case, symptoms started abruptly after the patient swallowed while bending over during dinner. The aetiology of non-traumatic laryngeal fracture is unknown. Pathological fracture due to an inflammatory condition or a neoplastic process should be considered [4]. However, in this case and all those reported previously, no underlying disease was found. One theory suggests the possible presence of a congenital anomaly or dehiscence of the cartilage, creating an area of weakness that is predisposed to fracture [2,6]. All previously reported cases occurred in males aged 29-47 years. They shared a common history of the patient feeling a crack in their neck abruptly after the precipitating event, before acutely developing dysphonia, dysphagia and odynophagia. All reported tenderness and swelling over the thyroid cartilage on examination. FNE findings included unilateral vocal cord oedema [4,5,7] and false cord haematoma [2,3,7]. Vocal cord movement was intact in all cases. Common findings on CT were of a non-displaced [3,4,6,7], or mildly displaced [5], anterior parasagittal laryngeal fracture. All patients were managed conservatively with observation and vocal rest, and made a full recovery.

This case gives support to there being a typical pattern to the presentation of this injury. Although all previous cases presented directly to the emergency department where same-day imaging allowed for a prompt diagnosis, this case highlights how this pathology may also be seen initially in primary care. In this setting, imaging and specialist review is not as readily available and thus can make diagnosis a challenge. This is the first reported case of a non-traumatic fracture being diagnosed upon re-presentation, where symptoms were rapidly evolving due to a large anterior neck swelling causing fracture displacement. While non-traumatic fractures appear to be frequently non-displaced and have previously all been amenable to conservative management, this case illustrates how concurrent adjacent infection and swelling can cause significant fracture displacement that requires operative intervention.

## Conclusions

Non-traumatic laryngeal fractures are rare; this case illustrates how they can pose a diagnostic challenge. It is the first to describe the presentation and surgical management of a case with fracture displacement due to localised infection. A typical history of a perceived sudden crack in the neck combined with the symptoms of dysphonia, dysphagia or odynophagia should raise suspicion and prompt further investigation for this pathology, even in the absence of a direct traumatic mechanism.
